# Stroke in the Adolescent Population

**DOI:** 10.15844/pedneurbriefs-34-3

**Published:** 2020-02-14

**Authors:** Ethan J. Rosenberg, Jonathan E. Kurz

**Affiliations:** 1 Division of Neurology, Ann & Robert H. Lurie Children’s Hospital of Chicago, Chicago, IL; 2 Departments of Pediatrics and Neurology, Northwestern University Feinberg School of Medicine, Chicago, IL

**Keywords:** Stroke, Ischemia, Adolescent, Revascularization, tPA

## Abstract

Investigators from 10 French academic centers studied a retrospective cohort of 60 patients aged 10-18 years (mean age 15.2 years) presenting with first-time stroke, as identified from discharge ICD-10 codes.

Investigators from 10 French academic centers studied a retrospective cohort of 60 patients aged 10-18 years (mean age 15.2 years) presenting with first-time stroke, as identified from discharge ICD-10 codes. These patients were treated in 10 adult and pediatric centers across 2 regions of France between 2007 and 2017. Subarachnoid hemorrhage or cerebral venous thrombosis were excluded from this study. Interestingly, the authors identified a significant proportion of their cohort with atheromatous risk factors more commonly associated with adult stroke, including cigarette smoking and obesity, although it is unclear how these rates compare to those of otherwise healthy adolescents. Among the underlying risk factors and conditions explored previously by the International Pediatric Stroke Study, chronic head and neck conditions, including migraine, were most common.

Although delayed diagnosis has been a significant problem in pediatric stroke, the majority of the patients in this study presented within 4.5 hours. 32% of patients were treated with hyperacute revascularization treatments, including IV tPA, intra-arterial thrombolysis, and mechanical thrombectomy. Patients presenting to adult hospitals were more likely to receive revascularization therapies. There were 3 instances of hemorrhagic transformation, one of which was symptomatic, and no symptomatic intracranial hemorrhages associated with revascularization treatments. There were no significant differences in 3-month outcomes with revascularization treatment, despite significantly higher initial NIHSS scores seen in these patients.

TOAST and CASCADE classifications were applied to each patient. 2 patients that were classified as undetermined in TOAST but were classified as unilateral arteriopathy according to CASCADE, possibly reflecting the focus on pediatric etiologies in this system. Recurrent cerebrovascular events occurred in 7 patients, all of whom had a vasculopathy based on CASCADE classification. [1]

COMMENTARY. Age-specific data to guide evaluation and management strategies is limited for adolescent stroke patients. In particular, the use of hyperacute revascularization therapies remain controversial [2]. Past efforts to study the safety and efficacy of tPA in children via a randomized controlled trial were unsuccessful, partially due to the medical comorbidities and diagnostic difficulties associated with pediatric stroke. Although the sample size is relatively small, this trial adds to retrospective evidence suggesting that revascularizing therapies may be safe, in this case in older children. Nearly one-third of the adolescents in this study were treated with endovascular therapies, with no symptomatic hemorrhages reported among those treated. This is consistent with other recent pediatric data from another small retrospective series [3].

As demonstrated by the data presented here, adolescents with stroke may share characteristics of both pediatric and young adults. This retrospective series is a good start to explore how these older children may experience unique risk factors and responses to treatment. Further study will rely on being able to consistently describe and categorize the etiology of stroke among this group. The results of this study would suggest that, despite their mixed characteristics, a pediatric-specific categorization schema remains the best option for adolescents. CASCADE is notable for its distinction of anatomic vascular etiologies and seems to be a more appropriate classification system in the adolescent population [4].

## Disclosures

The authors have declared that no competing interests exist.

## References

[1] Rambaud T, Legris N, Bejot Y, Bellesme C, Lapergue B, Jouvent E (2020). Acute ischemic stroke in adolescents. Neurology.

[2] Ferriero DM, Fullerton HJ, Bernard TJ, Billinghurst L, Daniels SR, DeBaun MR (2019). American Heart Association Stroke Council and Council on Cardiovascular and Stroke Nursing. Management of stroke in neonates and children: a scientific statement from the American Heart Association/American Stroke Association. Stroke.

[3] Amlie-Lefond C, Shaw DW, Cooper A, Wainwright MS, Kirton A, Felling RJ (2020). Risk of Intracranial Hemorrhage Following Intravenous tPA (Tissue-Type Plasminogen Activator) for Acute Stroke Is Low in Children. Stroke.

[4] Bernard TJ, Manco-Johnson MJ, Lo W, MacKay MT, Ganesan V, DeVeber G (2012). Towards a consensus-based classification of childhood arterial ischemic stroke. Stroke.

